# High-Frequency Local Field Potential Oscillations for Pigeons in Effective Turning

**DOI:** 10.3390/ani14030509

**Published:** 2024-02-03

**Authors:** Ke Fang, Xiaofei Guo, Yezhong Tang, Wenbo Wang, Zhouyi Wang, Zhendong Dai

**Affiliations:** 1 Institute of Bio-Inspired Structure and Surface Engineering, College of Mechanical and Electrical Engineering, Nanjing University of Aeronautics and Astronautics, Nanjing 210001, China; fangke@nuaa.edu.cn (K.F.); sz2205002@nuaa.edu.cn (X.G.); tangyz@cib.ac.cn (Y.T.); wwb523@nuaa.edu.cn (W.W.); 2 Chengdu Institute of Biology, Chinese Academy of Sciences, No. 9 Section 4, Renmin Nan Road, Chengdu 610041, China

**Keywords:** neural activity, higher-frequency oscillations, electrical stimulation, homing pigeons, turning behaviors

## Abstract

**Simple Summary:**

This study delves into the turning behavior of pigeons by examining the neural mechanisms of their midbrain motor nucleus. Correlating brain oscillations with turning behavior, we identified the distinct roles of oscillatory patterns in different frequency bands during active and passive turning behavior. Specifically, 80 Hz stimulation induced higher-frequency oscillation patterns. These findings unveil the intricate relationship between neural oscillations and pigeon turning, highlighting the significance of specific frequency bands. This study enhances our understanding of avian brain–behavior connections, offering valuable insights for further research on avian locomotion neural processes and serving as a reference for future studies on neuromodulation techniques in flying animal robots.

**Abstract:**

Flexible turning behavior endows Homing Pigeons (*Columba livia domestica*) with high adaptability and intelligence in long-distance flight, foraging, hazard avoidance, and social interactions. The present study recorded the activity pattern of their local field potential (LFP) oscillations and explored the relationship between different bands of oscillations and turning behaviors in the *formatio reticularis medialis mesencephali* (FRM). The results showed that the C (13–60 Hz) and D (61–130 Hz) bands derived from FRM nuclei oscillated significantly in active turning, while the D and E (131–200 Hz) bands oscillated significantly in passive turning. Additionally, compared with lower-frequency stimulation (40 Hz and 60 Hz), 80 Hz stimulation can effectively activate the turning function of FRM nuclei. Electrical stimulation elicited stronger oscillations of neural activity, which strengthened the pigeons’ turning locomotion willingness, showing an enhanced neural activation effect. These findings suggest that different band oscillations play different roles in the turning behavior; in particular, higher-frequency oscillations (D and E bands) enhance the turning behavior. These findings will help us decode the complex relationship between bird brains and behaviors and are expected to facilitate the development of neuromodulation techniques for animal robotics.

## 1. Introduction

Pigeons are renowned for their remarkable navigational and orienteering abilities, with homing instincts that enable them to return home over distances of thousands of miles [1,2]. These traits have captured the interest of scientists for a long time, as they not only illuminate the mysteries of animal behavior and biology but also provide new insights into artificial intelligence and navigation techniques [3,4,5,6]. However, delving into the biological basis of this navigational ability requires exploring the complex relationships between neural activity in the pigeon’s brain and the corresponding behavior.

Turning behavior is of importance in solving navigational challenges such as food searching, hazard avoidance, and homing in different contexts [7,8]. In the last few decades, relevant studies have made remarkable progress discovering a number of neural nuclei associated with turning behaviors in pigeons, by means of electrical stimulation methods. For example, electrical stimulation of the *dorsalis intermedius ventralis anterior* (DIVA), a somatic nociceptive area, or the *posterior pallial amygdala* (PoA), a fear-receptive area, can control left–right turning behavior in pigeons [9]. The same behavioral patterns can be obtained by stimulating the *formatio reticularis medialis mesencephali* (FRM), *nucleus rotundus* (RT), *occipitomesencephalic tractus occipito-mesencephalicus* (OM), and *nucleus tractus taeniae* (TN) in the midbrain motor area of pigeons [10,11]. However, it has been found that the responsiveness is stable when the sensory areas of the pigeon brain are excited electrically, and that these long-term negative stimuli may lead pigeons to exhibit non-adaptive physiological responses [12]. In contrast, stimulation of the midbrain motor nuclei can cause the typically stable initiation and execution of locomotion [13,14,15]. 

Given this, a comprehensive understanding of the neuromodulation mechanisms of turning behaviors in pigeons requires a study of the patterns of neural oscillatory activity within the relevant neural nuclei and their corresponding neural responsive patterns. The LFP is known to be an electrical signal that reflects the overall electrical activity of a localized population of neurons in the brain, and it can provide information about the neural activity of specific brain regions [16,17]. By recording the oscillatory activity of the LFP, the coordination and synchronization between brain regions—as well as their changes in specific tasks or behaviors—can be observed [17]. Previous studies have found that event-related oscillations in continuously recorded LFP signals are typically categorized into five frequency bands: delta (0.5–3.5 Hz), theta (4–7 Hz), alpha (8–12 Hz), beta (13–30 Hz), and gamma (31–80 Hz) [18,19]. Oscillatory activities in different frequency bands are closely related to various brain states and functions. They exhibit periodic rhythmic oscillations, act as universal operators or coding in functional brain activity, and have multiple functions [19,20]. Specifically, delta is the dominant frequency during deep sleep and is associated with learning, motivational processes, and brain reward systems [19,21]. Theta-band activity has been linked to working memory function, emotional arousal, and alertness [22,23]. Alpha band oscillations are believed to be associated with working memory functions and the maintenance of short-term memory, while beta-band oscillations are closely linked to sensorimotor functions and conscious decision-making [17,24,25,26]. Finally, oscillations in the gamma band may be closely associated with a variety of functions such as attention, working memory, sensory processing, action selection, and motor initiation and execution [27,28,29]. Each band of oscillations may be involved in the performance of multiple brain functions, and a single brain function may require the concerted involvement of oscillations in multiple different bands [19,20].

Most studies of neural oscillatory rhythms in the brain have focused primarily on mammals (humans, monkeys, rats, etc.) [18,30,31,32], with a relative paucity of studies in birds. Compared to mammals [30,31] and amphibians [33,34], birds exhibit unique patterns of electroencephalographic (EEG) features. Their EEG activity—especially high-gamma waves [35]—is usually more prominently characterized in the higher frequency range. It is noteworthy that although electrical stimulation has been recognized in a large number of mammalian studies as being able to activate, inhibit, or modulate neuronal activity [32,36,37] (which plays a crucial role in the brain’s nervous system), its application in avian studies has been relatively limited. In general, electrical stimulation methods can mimic natural neural activity or signaling in neural pathways, thereby triggering corresponding physiological and behavioral effects. At the same time, electrical stimulation can also intervene in neural activities and be used to study specific brain functions or neural mechanisms, to infer the role of neurons or neural pathways in specific physiological processes or behaviors. Therefore, an in-depth exploration of electrical stimulation in avian models is of crucial importance to reveal the specific nuances of neural oscillatory activity and its role in physiological processes and behaviors.

In the present study, we focused on the FRM nucleus in the midbrain motor area of Homing Pigeons (*Columba livia domestica*), which has been confirmed in previous studies to play a critical role in the control of walking turning and flight turning [10,38]. Based on this, our study hypothesized that pigeon turning motor behavior is related to neural oscillations in the FRM nucleus, and that neuroelectric stimulation can activate neural oscillations in different bands to participate in the modulation of turning behavior. To test this hypothesis, we recorded the activity patterns of LFP oscillations in the FRM nucleus and analyzed their absolute power spectra in different bands during turning. It can be expected that neural oscillations in different bands of the FRM nucleus of the pigeon brain represent different information processing or control pathways that can coordinate and modulate the turning behavior. The purpose of these studies is to improve our understanding of avian behaviors, especially in decoding the complex relationships between brain regions or circuits and behaviors, as well as to provide valuable references for further research on the neural mechanisms of bird navigation as well as the behavioral control of flying animal robots. 

## 2. Materials and Methods

### 2.1. Study Species

All Homing Pigeons were obtained from the loft on the roof of our building; each was born with an ankle-ring label indicating their date of birth. Twelve Homing Pigeons, aged 1–2 years (sex unknown), with a weight range of 428 g ± 25 g at the time of the experiment, were selected for this study. These subjects were housed in flocks in a loft on the roof of a building, kept under a typical diurnal light cycle, and ensured adequate food and water, which were freely available. All research on Homing Pigeons was conducted under the guidelines of the Chinese Regulations for the Management of Laboratory Animals and was approved by the Jiangsu Provincial Society for Laboratory Animals Scientific (Approval No. 2010012 and date of approval: 5 July 2010).

### 2.2. Surgery

**Figure 1:**
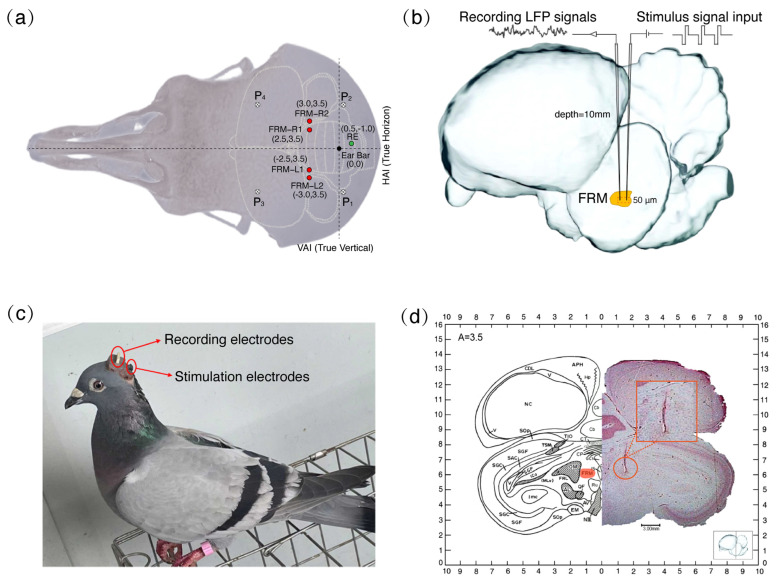
Electrode placement locations and a pigeon with implanted electrodes. (**a**) Coordinate positions of implanted electrodes in the *formatio reticularis medialis mesencephali* (FRM) nuclei of the pigeon brain. FRM−L and FRM−R represent the FRM nuclei regions of the left and right hemispheres. HAI and VAI correspond to the horizontal and vertical axes of the brain stereotactic apparatus, while RE denotes the reference electrode implanted above the cerebellum. P1, P2, P3, and P4 were connected to the ground wire and implanted above the lateral suture of the double parietal bone. (**b**) Depth of implantation of the stimulating electrode and the recording electrode, respectively (10 mm), and the spacing between the tips of the two electrodes (50 μm). (**c**) A pigeon implanted with stimulation and recording electrodes (ID: P05). (**d**) Coronal slices of the pigeon brain demonstrating that the electrode was implanted in the FRM nucleus.

The Homing Pigeons were fasted the day before the surgery. Electrode implantation was performed under general anesthesia (sodium pentobarbital 32 mg/kg, intramuscularly) supplemented by local anesthesia with 0.6 mL of lidocaine hydrochloride (0.5 mg/mL, subcutaneously in the surgical area). During the entire surgery procedure, the anesthesia statuses of the Homing Pigeons were determined by the toe-clamping response and supplemented with intramuscular pentobarbital (30% of the initial dose) if necessary. The pigeons were then fixed into a specially designed brain stereotaxic apparatus (Type 68027, RWD Life Science, Shenzhen, China) with the anterior fixation point (i.e., rostral bar position) located 45° below the horizontal axis of the apparatus. Using an aseptic technique, the dorsal surface of the skull was exposed; the cranial surface was cleaned with 3% hydrogen peroxide and rinsed with sterile saline before removal of the residual connective tissue. The FRM nuclei of the pigeon were selected as the only target nuclei, and the spatial coordinate positions of the FRM nuclei were determined from the homing pigeon brain atlas [39], with all stereotactic coordinates measured relative to the center of the ear rods and the cranial bone surfaces (Figure 1a,b). Each subject was implanted with stimulating and recording electrodes (paraformaldehyde-insulated nickel-chromium alloy wire, 100 μm diameter, Califonia Fine Wire, Grover Beach, CA, USA), with the tips of the two implanted electrodes spaced 50 μm apart. The electrodes were implanted in the FRM nuclei regions of the left and right hemispheres, and the other ends were soldered to different stimulating and recording electrode adapter plates. All electrode implantation edges were sealed with cyanoacrylate quick medical adhesive (EC) to seal the gap between the electrode and the skull. Four stainless steel screws (0.8 mm in diameter) labeled P1, P2, P3, and P4 were implanted on the cranial surface at a depth of about 5 mm under the skull for fixation of the electrode adapter plate, and silver wires were wound as the earth wire (Figure 1a). The reference electrode (RE) was buried in the cerebellum and implanted at a depth of approximately 10 μm. The electrode adapter plate on the cranial surface of the pigeon was fixed with dental acrylic resin after all electrodes were implanted, and the sockets on the electrode adapter plate were covered with self-sealing film (Parafilm M, Bemis Company, Chicago, USA) to avoid accidental occlusion. Subsequently, the pigeons were individually housed in (59 cm × 26 cm × 52 cm) wire cages (with adequate water and food) for a 6-day recovery period (Figure 1c), during which penicillin G was used to fight infection.

After all experiments were completed, five randomly selected pigeons were deeply anesthetized by injecting an overdose of sodium pentobarbital solution, then their brains were fixed through the sequential instillation of 75% saline and 4% formaldehyde solution. Subsequently, the brain was taken on a stereotaxic apparatus and subjected to histological analysis including sectioning and staining to confirm the correct positioning of the implanted electrodes on the FRM nuclei, in order to eliminate unexpected data (Figure 1d).

### 2.3. Behavioral Apparatus and Protocols

**Figure 2:**
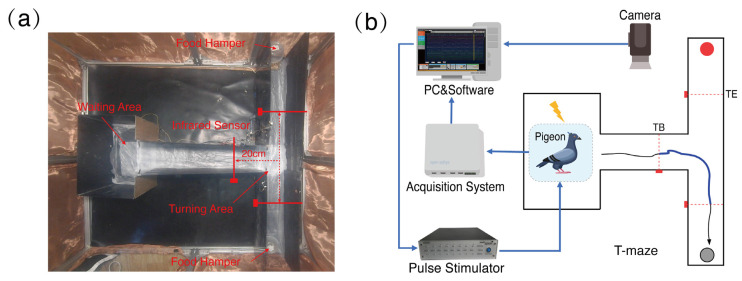
Behavioral experimental apparatus and experimental systems. (**a**) Customized T-maze apparatus for pigeons. (**b**) Overall system construction for turning behavior experiments in pigeons. TB: beginning of turning; TE: end of turning.

The subjects were placed on a restricted food supply of no less than 85% of their basal body weight for 7 days before the experiment in the T-maze, which was made of matte-black acrylic resin with 60 cm long and 20 cm wide lanes and 35 cm high walls. (Figure 2a). Two food boxes were located at the arm ends of the T-maze, and above each food box a switch was set for opening and closing by means of a fixed pulley device. Three infrared sensors (Type KGs-812A, Kegel, Guangdong, China) were mounted on the wall near the intersection of each of the three arms of the T-maze to monitor the entrance and exit of the turnstiles. When the pigeon was moved to the specified position, the infrared sensor was triggered and the TTL pulse signal was sent to the electrophysiological signal acquisition device in real-time via an Arduino development board, to synchronize the process of turning begin (TB) to turning end (TE) inside the maze. The entire T-maze setup was housed inside a specifically constructed Faraday cage (2.5 m × 2.5 m × 1.5 m) to avoid the effects of electromagnetic interference on the electrophysiological recording environment (Figure 2a). Simultaneously, a high-definition digital camera (Type RER-USB48MP02, Quan Rui Shi Xun, Shenzhen, China) was installed approximately 1 m above on the ceiling of the Faraday cage, for video recording of the corresponding behaviors. Each pigeon was subjected to a training protocol lasting 15–20 min per day, which forced the pigeons to learn to move freely within the T-maze and take food using food induction, with random placement of food to each arm each time until an 80% correctness was achieved.

### 2.4. Electrophysiological Recordings and Stimulation

**Figure 3:**
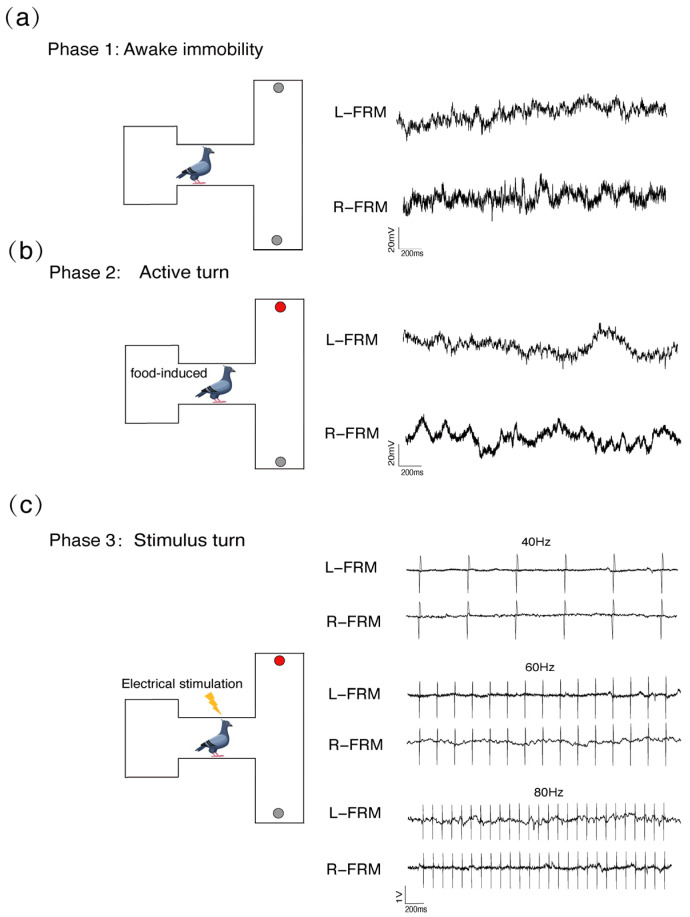
Experimental procedures and 2 s of typical local field potential (LFP) signal tracings for each channel. (**a**) Recording LFP signals in pigeons while awake and immobile. (**b**) Recording of LFP signals in pigeons during food-induced active turning. (**c**) Recording LFP signals of passive turning in pigeons at different electrical stimulation frequencies. L−FRM and R−FRM represent *formatio reticularis medialis mesencephali* (FRM) nuclei in the right and left brain hemispheres of pigeons, respectively.

About 24 h before the formal experiment, the subjects were connected to the signal acquisition–electrical stimulation system (Figure 2b) and placed in the T-maze for adaptive training [40]. The system was used to record the neurophysiological signals during the pigeon’s movement in the T-maze. Bandpass filtering was applied in the range of 0.05 to 500 Hz to obtain the LFP signals, and notch filtering at 50 Hz was employed to eliminate potential interference. The sampling frequency was set to 3 kHz. The signal for intracranial micro-stimulation was generated using a multichannel stimulation system STG-4008 (Reutlingen, Germany). Three constant-current pulse sequences with biphasic cathodic overdrive at three different frequencies of 40 Hz, 60 Hz, and 80 Hz (with the pulse width set to 1 ms and the stimulation duration set to 2 s were designed. The experimental design was divided into 3 main parts: LFP signals were recorded during the awake immobile state (AI) as a positive control (Figure 3a). They were also recorded during turning under free will (AT), as follows: the pigeons were placed in a T-shaped maze and induced to turn freely to trace foods; each pigeon was set to perform 100 steering trials in the left and right directions, during which the positions of the food boxes were randomly distributed with a 2-min interval between trials (Figure 3b). The LFP signals were recorded from FRM during turning under different electrical stimulations (Figure 3c), as follows: the pigeons were placed at the turning entrance of the T-maze, and the stimulus signals were applied when the pigeons were stationary. The stimulus signals were sent randomly to the left or right FRM in each trial for a total of 100 trials. To overcome the turning tendance attracted by food induction, the brain area which controls reversal turning orientation was stimulated.

### 2.5. Data Analysis

#### 2.5.1. Data Selection

In this study, all raw data extraction analyses were performed using a custom Matlab script (https://github.com/open-ephys/analysistools, accessed on 23 April 2023) (Matlab R2022b, The MathWorks, Inc., Natick, MA, USA). We synchronized the turning behavior and LFP data using the timestamps of the turning begin (TB) and turning end (TE) phases and calculated the average time taken to complete the active turning process (1.961 s ± 0.201) (Figure S1). Passive turning could be completed within the duration of the stimulation (2 s). Therefore, for the three different locomotor states of pigeons (awake immobile, active turning, and passive turning), we extracted the LFP data at 2 s before turning, during turning, and after turning in each trial for subsequent analysis.

#### 2.5.2. Stimulation Artifact Rejection

The raw signals of the LFPs recorded during stimulation were often accompanied by stimulation artifacts, which usually consist of two parts, the “instantaneous artifact spike” and the “artifact tail” [41]. The amplitude of the “instantaneous artifact spike” immediately following the stimulus pulse is usually more than 10 times the amplitude of the resting raw signal, reaching several hundred microvolts. In addition, these “artifacts” usually maintain a consistent shape for tens of milliseconds after each “instantaneous artifact spike” (Figure S2a). To remove the stimulus artifacts accompanying electrical stimulation, we drew on previous studies and employed a stimulus artifact suppression algorithm to eliminate these two artifacts [42,43].

The specific methods were as follows. (1) Thresholding the raw signals of LFPs recorded at the stimulation site to detect signal spikes. We used a window of 1.4 ms (from 0.6 ms before to 0.8 ms after the current artifact time) to extract “instantaneous artifact spikes”. Then, after each “instantaneous artifact spike”, we used another window to extract the “artifact tail” (Figure S2b). For the raw signals of the detected LFPs, we collected all “artifact tails” and categorized them according to the stimulus frequency. Then, for each category of “artifact tails”, we calculated its average value, which was used as a template for that category of “artifact tails”. (2) We excluded the “instantaneous artifact spike” portion of the raw data (the blue window bar portion) and considered this portion of the data as missing data because of its lack of utility. Typically, the duration of the missing data did not exceed 9% of the total stimulus duration. (3) To fill in the missing data, the −1.2 ms to −0.6 ms raw signal before the “instantaneous artifact spike” window (orange dashed window) was used to fill in the missing data from the first 0.6 ms to 0.0 ms. Similarly, the raw signal from 0.8 ms to 1.6 ms after the “instantaneous artifact spike” window (orange dashed window) was applied to fill in the missing data from 0.0 ms to 0.8 ms (Figure S2b). This interpolated substitution ensured that the amplitude and spectral distribution of the substituted raw data were similar to the background raw signal, thereby effectively removing the stimulus signal artifacts (Figure S2c) and guaranteeing the accuracy of the LFP signal [44].

#### 2.5.3. LFP Signal Preprocessing

After the removal of stimulus artifacts, the following pre-processing was performed on LFP signals in the three states: (1) downsampling of the raw LFP signals to 512 Hz; (2) offline filtering of the LFP signals using a 1–200 Hz band-pass filter, to remove potential high-frequency noises including motion activity; (3) removal of linear trends in the LFP signals using a least-squares fitting algorithm; (4) deletion of power-supply noise interference using a 50 Hz notch filter; (5) for LFP signals recorded during stimulation, discarding of possible residual stimulus artifacts at the stimulation frequency using comb filters of different frequencies. Subsequently, we referenced the LFP signal of each channel to its nearest-neighboring stimulated channel according to the location and depth of electrode implantation, removing channels exhibiting noises during stimulation (standard deviation of the LFP signal during stimulation > 5 times that before stimulation).

After obtaining the pre-processed LFP signals, we investigated the spectral characteristics of the LFP signals using power spectral density (PSD) analysis. Specifically, the Welch method was used and a Hamming window (1.0 Hz resolution) was chosen to calculate the absolute power spectrum of the LFP signal during turns, as well as to extract each featured band in the signal [45,46]. In addition, we employed time–frequency analysis (TFA) of the Morlet wavelet transform to analyze the change pattern of the spectral characteristics in the LFP signal over time [47]. Previous neurobehavioral studies have shown considerable differences in rest-activity patterns of electrophysiological features across animal species. Five characteristic frequency bands were extracted based on previous studies in pigeons: A: 0.5–3 Hz; B: 4–12 Hz; C: 13–60 Hz; D: 61–130 Hz; and E: 131–200 Hz (Figure S3). Considering that the range of intervals we describe is not consistent with the range studied in mammals, we avoided using Greek letters to name the bands. For each subject, the mean absolute power spectra of the five frequency bands were calculated and log-transformed for further statistical analyses.

### 2.6. Statistical Analyses

Prior to statistical analyses, the absolute power spectral values of the LFP for 12 pigeons under different behavioral parameters were tested for normal distribution and homogeneity of variance using the Shapiro–Wilk *W* test and the Levene’s test, respectively. Given that these LFP data satisfied the statistical assumptions of normal distribution and homogeneity of variance, a three-factor repeated-measure ANOVA with “stimulus”, “bands”, and “brain region” as factors was used in the subsequent statistical analyses to analyze the main and interaction effects among the factors in different behavioral states. Moreover, if the interaction was significant, further simple effects analysis or simple-simple effects analysis was performed. The least significant difference (LSD) was used for post hoc tests to analyze the data for multiple comparisons. Greenhouse–Geisser correction was applied for failure to meet the test of sphericity; effect sizes were detected by partial *η*^2^ (0.20 for low effect sizes, 0.50 for medium effect sizes, and 0.80 for high effect sizes) [48]. All analyses were conducted using IBM SPSS Statistics 26.0 (IBM Corporation, Armonk, USA) and *p*-values were marked as statistically significant as follows: * *p* < 0.05, ** *p* < 0.01, and *** *p* < 0.001 [49].

## 3. Results

**Figure 4:**
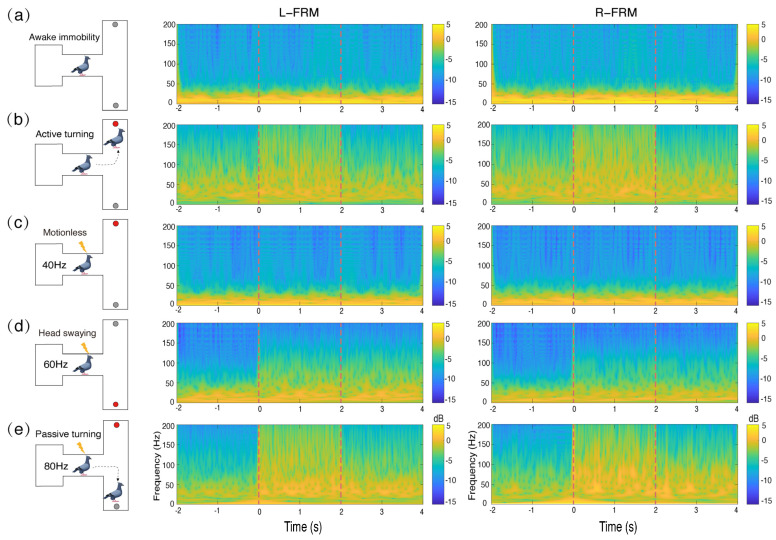
Results of behavioral responses (**left**) and time–frequency plots of local field potential (LFP) oscillations (**right**) of pigeons under different stimulus conditions: (**a**) the awake and motionless state of the pigeon, (**b**) the active turning state of the pigeon, (**c**) the motionless state of the pigeon in response to the 40 Hz stimulus, (**d**) the slight head bobbing state of the pigeon in response to the 60 Hz stimulus, and (**e**) the passive turning state of the pigeon in response to the 80 Hz stimulus. Gray circles represent food boxes without food, red circles represent food boxes with food; yellow arrows are symbols of electrical stimulation; L−FRM and R−FRM represent *formatio reticularis medialis mesencephali* (FRM) nuclei in the right and left brain hemispheres of pigeons, respectively.

The behavioral responses of pigeons under different stimulus conditions induced LFP oscillatory activity in their midbrain FRM nuclei regions with specific time–frequency characteristics (Figure 4). With electrical stimulation at 40 Hz, pigeons remained motionless (Figure 4c). At this time, the time–frequency characteristics of the LFP oscillations were highly similar to those of its awake immobile state, in which oscillation frequencies were below 25 Hz (Figure 4a,c). Moreover, during the 40 Hz micro-stimulation period, no significant changes in the LFP spectral oscillations in either the right or left brain regions of the FRM were observed (Figure S4). While using the 60 Hz electrical stimulation procedure, the pigeons exhibited a slight head swaying, despite the lack of observed turning behavior. The head swaying oriented to the side on which the brain region was stimulated (Figure 4d). There was a difference in the time–frequency characteristics of the LFP oscillations observed between the awake immobile state and that under 40 Hz stimulation. In periods before and after 60 Hz stimulation, we observed a certain degree of LFP oscillatory activity in the pigeon FRM nuclei region, with the oscillatory frequency range approximately widened to 0–60 Hz (Figure 4d and Figure S4). This oscillatory activity induced by stimulations continued for 2 s after the stimulation stopped.

The pigeons exhibited a strong passive turning behavior with 80 Hz stimulation in the FRM nuclei. The stimulation was so effective that the pigeons could overcome their foraging instincts by turning in the opposite direction to the food box (Figure 4e). Compared to the food-induced condition, LFP oscillatory activities in the FRM nuclei were significantly increased when they performed either active or passive turning with 80 Hz stimuli. The spectral oscillatory patterns, mainly centered in the 60–200 Hz range, differed largely from those in the resting condition (Figure 4b,e). Similar spectral oscillatory activity could be observed in the contralateral FRM nuclei regions, regardless of whether the left FRM nuclei region or the right FRM nuclei region was stimulated (Figure S4).

**Figure 5:**
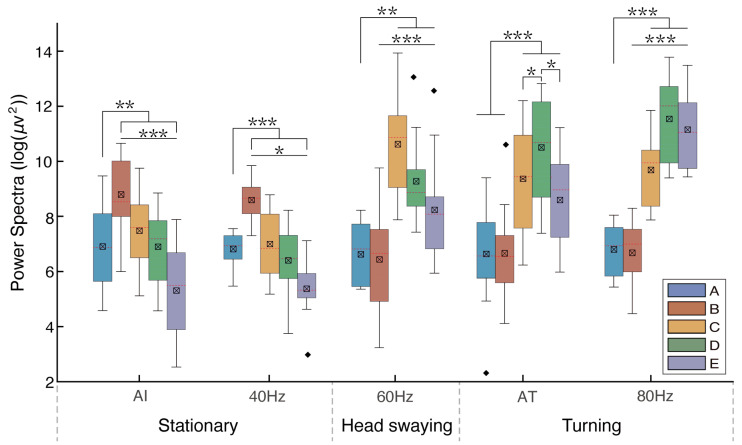
The mean absolute power spectra of pigeons across various behavioral states, with A, B, C, D, and E representing five different characteristic bands. The least-significant difference test (LSD) was employed for conducting pairwise comparisons between these different bands. Abbreviations: AI: pigeons’ awake immobile state; AT: pigeons actively turning, induced by food. The values 40 Hz, 60 Hz, and 80 Hz represent the different frequencies of electrical stimulation. Among them, at 80 Hz, pigeons showed passive turning behavior. Each asterisk indicates significant and highly significant differences (* *p* < 0.05, ** *p* < 0.01, and *** *p* < 0.001) in the mean power spectrum between different stimuli.

Multiple comparisons indicated that the absolute power spectra of the LFP oscillations were significantly greater during turning locomotion (AT; 80 Hz) and head bobbing (60 Hz) than those in the awake immobile state (AI; 40 Hz) (Table 1 and Figure 5). For immobile pigeons, the B band of the power spectra was significantly larger than the A, C, D, and E bands (Table 1 and Figure 5). When stimulated at 60 Hz, significantly higher power spectral values were observed in the C band compared to the A, B, D, and E bands. Subsequently, the C, D, and E bands exhibited significantly greater power than the A and B bands when pigeons engaged in active turning behavior following food induction. Notably, band D showed significantly higher power compared to bands C and E. When pigeons performed passive turning in response to the 80 Hz stimulus, the C, D, and E bands showed significantly higher power values than the A and B bands. In addition, the D and E bands exhibited significantly greater power than the C band, with the D band being significantly larger than the E band. Notably, during active turning, neural activity in the C and D bands demonstrated a preferential increase, whereas in the context of passive turning, neural activity in the D and E bands exhibited a preferential enhancement.

**Figure 6:**
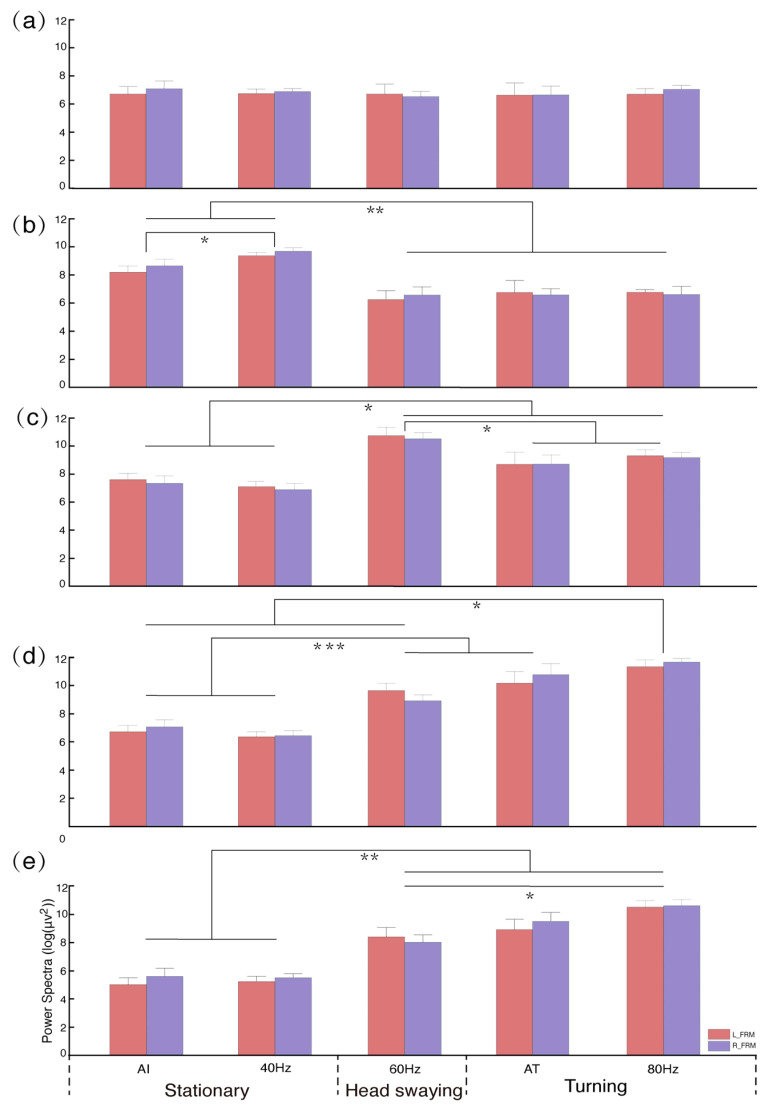
Absolute power spectra in the *formatio reticularis medialis mesencephali* (FRM) nuclei regions of the left and right hemispheres for different stimulus conditions: (**a**–**e**) the five different characteristic bands. The least-significant difference test (LSD) was employed for conducting pairwise comparisons between these different stimulus conditions. Abbreviation: AI: pigeons’ awake immobile state; AT: pigeons actively turning. The values 40 Hz, 60 Hz, and 80 Hz represent the different frequencies of electrical stimulation. Asterisks denote significant and highly significant differences (* *p* < 0.05, ** *p* < 0.01, and *** *p* < 0.001).

The FRM nuclei, a pivotal brain region responsible for turning functions in pigeons, exhibits five distinct characteristic frequency bands of the LFP, each manifesting unique oscillation patterns in response to various stimuli. There were no significant disparities between the left and right hemispheres of the FRM (*p* > 0.826) (Figure 6 and Figure S5, Table S1). Conversely, within the B band, the mean power spectrum of pigeons in the stationary state was significantly greater than that involved in directional head bobbing and turning locomotion (Figure 6b and Table S1). The C band showed significantly higher mean power spectra during directional head bobbing and turning locomotion than when motionless, and that induced during directional head bobbing was significantly greater than that during turning locomotion (Figure 6c and Table S1). For the D band, the mean power spectrum during passive turning locomotion was significantly higher than those in a motionless state (AI, 40 Hz) and during directional head bobbing. The E band from turning locomotion was significantly higher than that in other behavioral states (head bobbing and motionless). Intriguingly, passive turning locomotion was observed to yield a significantly higher power compared to active turning (Figure 6e and Table S1).

## 4. Discussion

### 4.1. LFP Oscillations in FRM Nuclei and Turning Behaviors in Pigeons

Our study reveals that neural oscillations in different frequency bands in the FRM nuclei of the pigeon brain have specific functional and physiological significance. Low-frequency neural oscillations (A and B bands) dominate when pigeons remain awake and immobile. However, in the modulation of their turning behavior, oscillations in the middle and high frequencies (such as the C, D, and E bands) show an important activation role. Recent studies have shown that neuronal oscillations in different frequency bands are considered important for the synchronization of neuronal assembly, binding, and plasticity [50]. These oscillatory phenomena are regarded as universal operators or coding modalities for functional brain activity and are therefore involved in a wide range of physiological functions [19,20]. According to previous studies on the spectra of electroencephalographic (EEG) features in pigeons during sleep and wakefulness, the oscillation pattern of electrophysiological features in the low-frequency band A (0.5–3 Hz) is similar to that of delta waves (0.5–4 Hz) in mammals (e.g., rats, cats, and humans, etc.) and belongs to the high-amplitude slow waves [32,51,52,53,54]. These oscillations are associated with deep sleep, unconsciousness, or certain pathological states. In avian species, delta waves usually occur during deep rest or sleep [52,55]. B band (4–12 Hz) had a significantly higher mean power spectrum in the awake immobile state in our study, due to the fact that the B band in the domestic pigeon is functionally equivalent to the theta wave (3–12 Hz) in mammals, which typically oscillates during alert immobility, voluntary movement, and rapid eye movement (REM) sleep. In mammals (e.g., rabbits, guinea pigs, rats, and cats), the theta wave rhythms occur in the complete absence of locomotion (referred to as type 2 theta) and are commonly associated with spontaneous locomotor behaviors such as alert immobility, chewing, licking, and grooming [56,57,58,59]. Combining this with our results, we see that the pigeons may have an electrophysiological state of “alertness and exploration” similar to that of mammals when they are awake and immobile. Thus, despite the obvious evolutionary differences between birds and mammals, this rhythmic slow-wave activity theta wave may be an ancestral property of the brain’s nervous system that has been preserved throughout vertebrate evolution, allowing the theta-wave oscillations to exhibit similar functional patterns.

In contrast, oscillatory activation in the C, D, and E bands of the FRM brain area plays an important role in modulating turning locomotion in pigeons. Previous studies have demonstrated that in pigeons, the C band (13–60 Hz) corresponds to the beta wave (12–30 Hz) in mammals [35,60]. The beta wave, characterized by low amplitude and fast frequency, has long been associated with sensorimotor integration, coordination, motor preparation, and attention. Additionally, the D band (61–130 Hz) in pigeons is analogous to the gamma wave (30–80 Hz) in mammals. The gamma wave, a relatively high-frequency brain wave, plays a crucial role in coordinating and synchronizing activities between different brain regions, serving as a key component in brain information integration and processing [28,29]. The E band (131–200 Hz) is considered to be the counterpart of the high-gamma (80–100 Hz) wave, which belongs to the higher-frequency portion of the gamma wave and is commonly associated with higher cognition and decision-making, playing a key role in the transfer of information between different regions in the brain [28,35,61]. It has been demonstrated that differences in the EEG frequency ranges of birds and mammals can be partially attributed to their neurological structure and lifestyle adaptations. 

The bird brain has a smaller cortex and a different functional layout, with a brain structure that is more focused on functions such as vision and motor control [62]. This may result in a higher range of EEG frequencies in birds, and higher frequencies may be better suited for processing fast visual information and coordinating precise movements. In the present study, the pigeon’s C band was consistent with a pattern of beta-wave oscillations, with significant oscillatory activation manifested during both the pigeon’s active turning and head bobbing states. Recent evidence supports the role of the oscillatory activity of beta waves as a general coupling mechanism for neuronal activity within and across brain structures, with discrete beta wave oscillations occurring almost simultaneously in upstream and downstream nuclei when motor behavior occurs [63,64,65]. This state of elevated beta-wave power reflects not just sensory processing or motor output but the state of the subject when using sensory cues to determine voluntary behavior. In general, beta-wave oscillatory activity increases significantly when the animal explicitly engages in a specific task, whereas beta-wave oscillations are not significant during spontaneous behavior [26,66]. In addition, several studies have confirmed that beta-wave oscillatory activity in the motor cortex increases in primates after receiving guided motor cues and decreases during actual motor execution [63,65]. In addition, several studies have confirmed that beta-wave oscillatory activity in the motor cortex increases in primates after receiving guided motor cues and decreases during actual motor execution. Meanwhile, in studies of goal-directed behavior in pigeons, it was found that pigeons oscillate in their hippocampal and NCL brain regions in the frequency range of 40–60 Hz when they are at the turn of a maze [67,68]. Combining this with the present results, we found that oscillatory activity in the C band was significantly increased during the execution of an active turning task in pigeons and was higher than during their awake immobility state. While the pigeon’s head bobbed slightly (60 Hz stimulation) (possibly an activation of a guided motor cue), the C-band oscillations were again significantly higher than those when executing the turning task. 

Notably, the average power spectrum in the D band of the pigeon FRM brain region exhibited maximal values in both active and passive turning states. It is well known that gamma oscillations are caused by interactions between interconnected inhibitory interneurons and pyramidal cells, and that such interactions can establish synchronization and coordination mechanisms between different motor control regions of the brain, which helps to achieve smooth body movements [69,70]. Meanwhile, gamma oscillatory activity in the motor cortex is thought to essentially facilitate movement because gamma oscillations establish connections between the sensory cortex and the motor cortex, which, in turn, match sensory feedback (e.g., visual and tactile information) with motor execution, ensuring that the body responds accurately during movement [28,71]. In particular, frequency-specific increases in gamma oscillations are positively correlated with their activation strength, which provides further evidence for a causal relationship between gamma activity in the motor cortex and motor behavior [71,72]. Consistent with this, pigeons did not show significant oscillations in the D band when they were stationary, but did show them when they were turning during locomotion, which suggests that the D band has a critical role in modulating turning locomotion. Furthermore, it was interesting to note that the E band tended to oscillate significantly only when pigeons were turning passively. The oscillatory activity in high-gamma waves usually might be involved in synchronized activity between multiple neuronal populations. In motor control, these neuronal populations can include regions that play key roles in motor execution, sensorimotor feedback, and motor planning, and they help the brain to adapt and respond to the demands of motor tasks at different hierarchical levels [28,73]. Simultaneously, the significant oscillations in the E band may reflect the activation of more advanced sensorimotor feedback mechanisms from the triggering of a passive turning behavior.

### 4.2. Neural Associations between Oscillation Bands in Different Turning Behavior Patterns

The activities of EEG frequency bands reflect the activity states of the brain under different information processing or control pathways, and these bands reflect different types of neuronal activities and neural network interactions, as well as their roles in different cognitive and behavioral tasks [50,74]. Specifically, oscillatory activities of the C and D frequency bands showed significant enhancement during active turning, whereas oscillatory activities of the D and E frequency bands were more prominent during passive turning. This suggests that different turning locomotion patterns are modulated by different neural oscillatory frequency bands.

First, active steering may require more oscillations in the C and D bands to support motor planning and execution, which is consistent with the need for more brain control and coordination in active behavior. Previous studies in humans and monkeys have shown that active control requires flexibility to adapt behavior to changing environments [75,76]. When performing motor tasks, subjects are not just driven by triggers triggered by external stimuli; rather, their behavior needs to be guided and controlled by internal signals or internal mechanisms [75,76]. This may involve various regions of the brain and neural circuits responsible for the perception, decision-making, planning, and execution of actions. In the present study, active turning was primarily driven by food induction, requiring pigeons to actively engage in motor planning and control to obtain food. This behavior involves the inter-coordinated action of multiple brain regions in pigeons and relates to the pigeon’s active willingness, decision processing, and motor control. Moreover, it has been shown that beta and gamma oscillations were frequently observed in motor-related brain circuits during motor preparation and execution, and that there was a mutual coupling between them. This coupling relationship supports higher cognitive functions through the transfer of information and coordination between different brain regions, leading to more efficient processing and the integration of information from different sensory sources [77,78]. The coupling of beta and gamma bands may enhance synchronization between neural networks that play a role in coordinating and performing fine motor tasks [78]. Thus, it is reasonable to believe that this coupling is present and has an important role in the active turning of pigeons.

In contrast, passive turning in pigeons may be more dependent on oscillations in the D and E bands, which may be related to the fact that passive locomotion is generally modulated by sensory feedback and responses and does not require the involvement of an active motor program. In the present study, pigeons overcame their instinctive responses and underwent turning locomotion in the direction of the brain stimulus when subjected to an electrical stimulus of 80 Hz. This means that the external stimulus mandatorily changed the direction of their locomotion without the involvement of active decision-making. Previous studies in humans have demonstrated the existence of neural coupling mechanisms between gamma and high-gamma waves in motor control behaviors, especially between different regions of the brain cortex [72]. The frequency of these waves is associated with different aspects of motor control, coordination, and perceptual–motor integration [27,29]. The study notes that gamma waves were associated with the firing activity of individual motor cortex neurons, especially during motor planning and execution, whereas high-gamma waves were generally associated with finer motor control and perceptual–motor integration, especially during motor tasks requiring fine coordination [72]. Recent studies have also pointed out that in the visual cortex of humans and rhesus monkeys, gamma and high-gamma waves typically respond rapidly under different visual stimulus conditions to participate in the transmission and integration of neural information [70,79]. Similarly, in studies of spatial path tuning in pigeons, it has been found that as pigeons adjust the spatial path of their movements, the functional network connectivity of their hippocampal and NCL brain regions is selectively altered, with decreasing connectivity in the lower bands (delta and theta) and elevated connectivity in the higher bands (gamma and high-gamma) [80,81]. Combining this with the present study, we see that FRM regions may need to process sensory feedback and rapid responses to external stimuli more rapidly during passive turning in pigeons, which may lead to a significant increase in D and E band oscillations.

Overall, these different brainwave oscillation patterns may reflect the neural activity and behavioral demands of pigeons under different conditions. During active turning, pigeons may be more focused on perception and fine motor control, hence the coupled oscillations in the C and D bands. Meanwhile, during passive turning, rapid sensory feedback and external stimuli may lead to a significant increase in D and E band oscillations for rapid adaptation to external stimuli.

### 4.3. Specific Modulation of Turning Behavior and Neural Oscillations by Electrical Stimulation

In the present study, different electrical stimulation frequencies produced differential electrical stimulation effects on the motor behavior of pigeons and the characteristic frequency-band oscillation patterns of LFPs in the FRM brain region, thereby affecting the electrical activity patterns of the pigeon brain and its motor behavior. This result is consistent with recent studies that electrical stimulation in a frequency-specific manner can modulate the electrical oscillatory rhythms of the brain, thereby modulating its motor and cognitive functions to a certain extent [32,36,37,82,83]. In general, high-frequency stimulation usually results in neurons being more excitable, while low-frequency stimulation may inhibit neuronal activities. Meanwhile, stimulation at different frequencies can modulate neuronal synchronization and coherence, and these properties are critical for information transmission and processing. When electrical stimulation at 60 Hz was applied, the pigeons did not show significant turning locomotion but only slight head bobbing, and the C band oscillations were significantly enhanced. By stimulating this area, the signaling of neural circuits can be influenced, thereby adjusting or mimicking the neural circuits associated with behavior or function, directing the animal to perform a different behavior [84,85,86]. 

Notably, the pigeons exhibited passive turning locomotion when electrically stimulated at 80 Hz. The oscillatory activity was significantly enhanced during passive turning, mainly in the D and E bands. Active locomotion requires a higher degree of neural control and coordination, hence the enhanced oscillations in the C and D band; meanwhile, passive locomotion in response to 80 Hz stimulation may involve more reflexive control, hence the more pronounced oscillatory activity in the D and E bands. This phenomenon may be related to the enhanced neural activation effects of electrical stimulation. Previous studies have demonstrated that the neural activation effect enhanced by electrical stimulation may involve multiple neural mechanisms, including synaptic strengthening and neuronal synchronization [37,87]. Stimulation frequency can affect synaptic plasticity, and low-frequency stimulation may lead to long-duration synaptic inhibition, whereas high-frequency stimulation can either enhance or inhibit connections between neurons, thereby altering signaling patterns and activating potential neural pathways [87,88]. These studies also confirm our findings that the frequency of electrical stimulation can trigger neural oscillatory activity in different bands, sometimes inducing stronger oscillations of neural activity and enhanced neural activation effects, which will reinforce specific behavioral responses.

### 4.4. Study Limitations

Homing Pigeons have the ability to move freely in three-dimensional space, and their turning behavior involves not only turning when walking on the ground but also turning during natural flight. Pigeons turn mainly through leg movements on the ground, whereas in natural flight, turning is mainly accomplished through coordinated wing movements. In the present study, selected FRM nuclei in the midbrain motor area in Homing Pigeons have been shown to be important in both walking and flight turning [10,38]. However, the limitations of the experimental setting led to these results being based primarily on pigeons turning while walking on the ground rather than natural flight turning. However, according to existing studies, the higher nervous system of animals is able to sense changes in the locomotor environment and execute different motor neural circuits and motor actuators (e.g., legs or wings) through the midbrain motor nucleus, to control turning behaviors in different environments (walking and flight states) [13,15]. This neural response usually shows some consistency across environments and states. Thus, the neural circuits as well as the motor control systems for flight turning and walking turning in pigeons have evolved to share many similar basic features, and we strongly believe that these patterns of neural oscillations represent a more general neural mechanism that plays a critical role in this process.

In the future, we will consider conducting experiments under flight conditions that are closer to natural conditions. This will allow us to verify the relationship between neural oscillations and turning behavior in pigeons, while also gaining a more comprehensive understanding of the mechanisms involved in their neurobiology and locomotor behavior. 

## 5. Conclusions

This study investigated the impact of varied stimulation frequencies on LFP oscillatory activity in the FRM nuclei region and turning motor behaviors in pigeons. Spectral analysis revealed that oscillatory activities in the C, D, and E bands within the FRM nuclei play a crucial role in modulating pigeon turning behavior. Mutual coupling between these bands influenced distinct turning locomotion patterns, with 80 Hz stimulation notably enhancing high-frequency oscillatory patterns and improving turning behavior. The findings highlight the nuanced role of neural oscillations in avian brain function, suggesting that different frequency bands represent distinct information processing pathways. Furthermore, this study further confirmed that electrical stimulation can effectively modulate neural oscillations in the pigeon’s brain and significantly affect its turning locomotion function. This finding provides substantial guidance for the design of future electrical stimulation systems for animal robots, and the use of these neural oscillation patterns can optimize electrical stimulation parameters to enable more precise control of an animal robot’s locomotor behaviors, which can be more closely aligned with real natural behaviors.

## Figures and Tables

**Table 1:** Results of three-way repeated-measure ANOVA for the factors “Brain area”, “Stimulus”, and “Bands” for the five local field potential (LFP) bands.

Factor	*F*_(1, 22) (4, 44) (4, 44) (16, 176)_ ^a^	*ε*	*p*	Partial *η^2^*	LSD
Brain area	0.050	N/A	0.826	0.002	N/A
Stimulus	7.633	0.704	<0.001	0.410	AT, 60 Hz, 80 Hz > AI, 40 Hz
Bands	39.591	0.481	<0.001	0.783	B, C, D, E > A; C, D > B, E
2-way interaction	29.570	0.301	<0.001	0.729	Bands X stimulus (see Table S1)
					Stimulus X bands
Stimulus _(4, 8)_|(AI, Bands)	16.889	N/A	0.001	0.894	B > A, C, D, E; A, D > E; C > D
Stimulus _(4, 8)_|(AT, Bands)	37.936	N/A	<0.001	0.950	C, D, E > A, B; D > C, E
Stimulus _(4, 8)_|(40 Hz, Bands)	74.049	N/A	0.002	0.850	B > A, C, D, E; A, C, D > E
Stimulus _(4, 8)_|(60 Hz, Bands)	11.312	N/A	0.002	0.850	C > A, B, D, E; D, E > A, B; D > E
Stimulus _(4, 8)_|(80 Hz, Bands)	37.837	N/A	<0.001	0.950	C, D, E > A, B; D, E > C; D > E

Note: The symbol ‘X’ denotes that the factor between the “stimuli” and “bands” has an interaction effect. The superscript symbol “^a^” in the first line of the table denotes the degrees of freedom for the factors “Brain area”, “Stimulus”, and “Bands”, respectively. The numbers in parentheses indicate the values of the degrees of freedom of the factors. *F* is the *F*-value from ANOVA, *ε* denotes the values of epsilon of the Greenhouse–Geisser correction, LSD denotes the least-significant difference test; and A, B, C, D, and E represent five different bands. Abbreviations: AI: the pigeons’ awake immobile state; AT: pigeons actively turning, induced by food. The values 40 Hz, 60 Hz, and 80 Hz represent the different frequencies of electrical stimulation. N/A not applicable.

## Data Availability

The original contributions presented in the study are included in the article/Supplementary Material, further inquiries can be directed to the corresponding authors.

## Supplementary Materials

The following supporting information can be downloaded at https://www.mdpi.com/article/10.3390/ani14030509/s1: Figure S1: Mean time for pigeons to actively turning when induced to do so by food. Figure S2: Removal method of electrical stimulation artifacts. Figure S3: Extraction of different feature frequency bands in *formatio reticularis medialis mesencephali* (FRM) nuclei regions. Figure S4: Time-frequency maps of electrically stimulated *formatio reticularis medialis mesencephali* (FRM) nuclei regions and their LFP oscillations on different sides of the pigeon. Figure S5: The average waveforms of power spectral density (PSD) for each *formatio reticularis medialis mesencephali* (FRM) nuclei area and each stimulus conditions. Table S1: Results of the simple-simple effects analysis between the five different characteristic bands of local field potential (LFP) and the stimulus conditions in *formatio reticularis medialis mesencephali* (FRM) nuclei regions.
