# Supplementary material for: High-Frequency Local Field Potential Oscillations for Pigeons in Effective Turning
Source: Animals (Basel). 2024 Feb 3;14(3):509. doi: 10.3390/ani14030509 (PMC10854807; doi:10.3390/ani14030509)
Supplement: Supplementary file 1 [file animals-14-00509-s001.zip › animals-2769554-supplementary.pdf]

Supplementary Materials

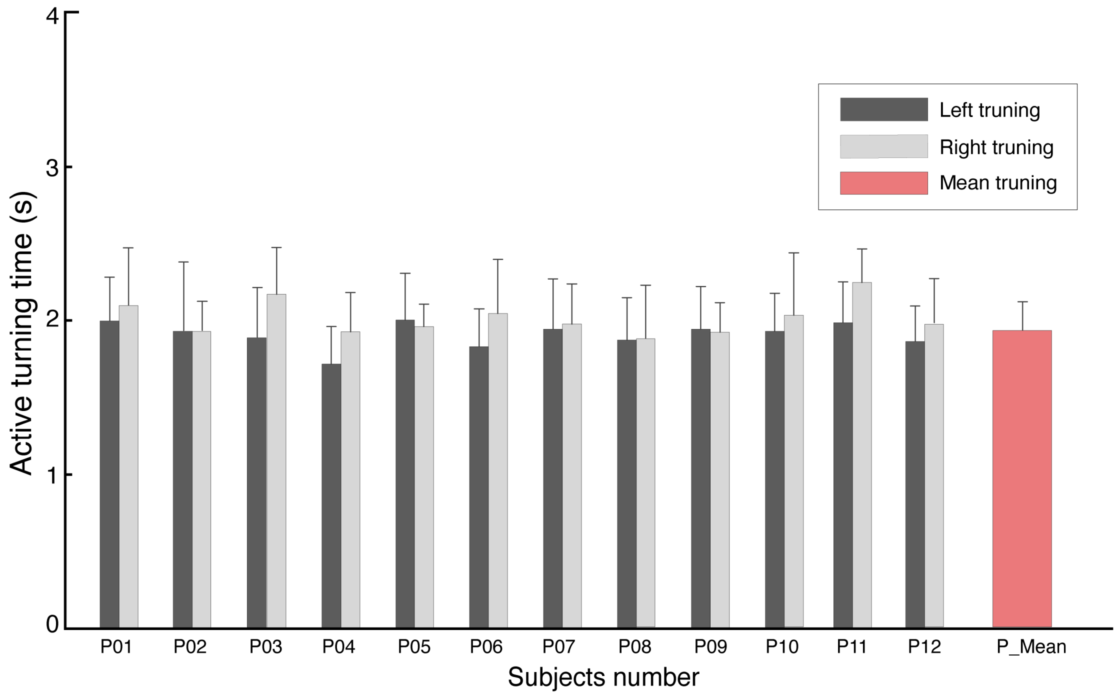

Figure S1. Mean time for pigeons to actively turning when induced to do so by food.

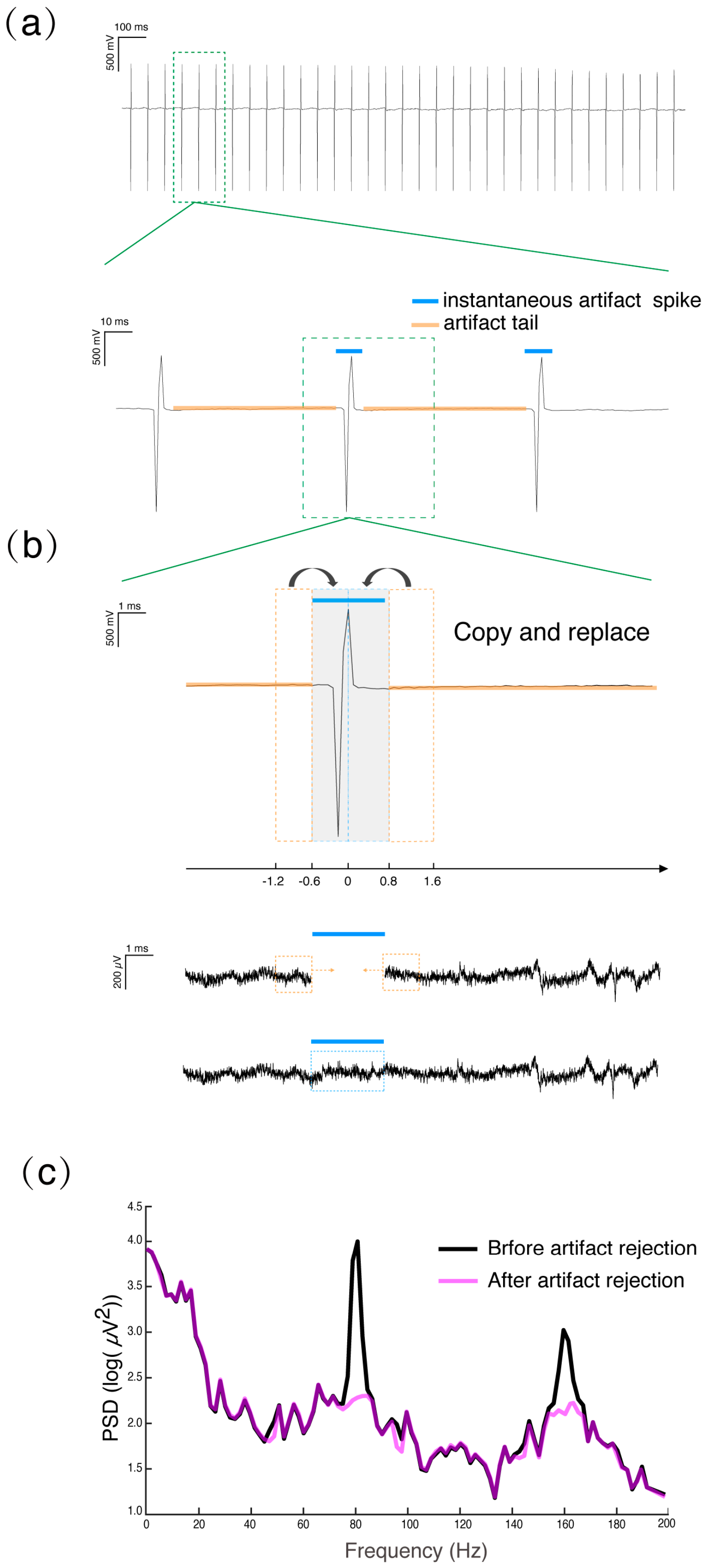

Figure S2. Removal method of electrical stimulation artifacts. (a), An example channel shows the procedure of stimulation artifact rejection. Raw signal recorded during stimulation was masked by large stimulation artifacts consisting of a large “instantaneous artifact spike” and a

long “artifact tail”. (b), Obtaining the template of the “artifact tail”. Orange bars represent the time window for obtaining the template, which was chosen as the ending point of the current “instantaneous artifact spike” to the starting point of the next “instantaneous artifact spike”. The orange signal represents the obtained template of the “artifact tail”. Removal of the “artifact tail” using template subtraction and removal of the “instantaneous artifact spike” using interpolation. The “artifact tail” template (orange) was subtracted from the raw signal. The data during the “instantaneous artifact spike” window (blue bar) was discarded and treated as missing data. The missing data was then interpolated by the raw data immediately before and after the “instantaneous artifact spike” window (dashed orange boxes). (c), Power spectral density (PSD) of the stimulation artifact-rejected signal compared with the raw signal.

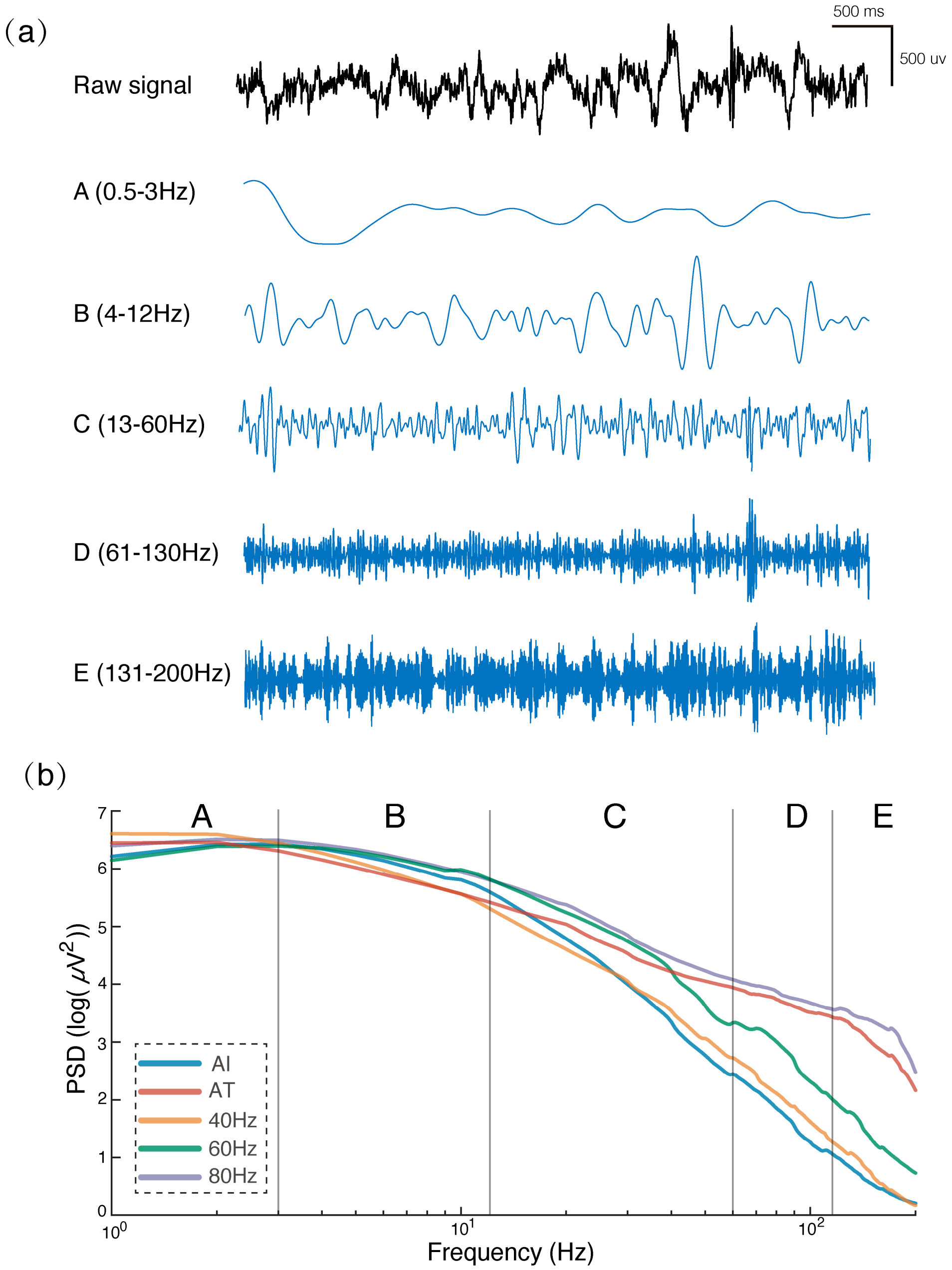

Figure S3. Extraction of different feature frequency bands in *formatio reticularis medialis mesencephali* (FRM) nuclei regions. (a) Examples of simultaneous local field potentials recorded raw signal from the FRM nuclei and filtered at A, B, C, D, E frequency bands. (b) Mean power spectrum curves under different stimulus conditions corresponding to different bands (A, 0.5–3 Hz; B, 4–12 Hz; C, 13–60 Hz; D, 61–130 Hz; E, 131–200 Hz). Abbreviation: AI represents pigeons' awake immobility; AT represents pigeons actively turning induced by food. 40 Hz, 60 Hz, and 80 Hz represent the different stimulus conditions of electrical stimulation.

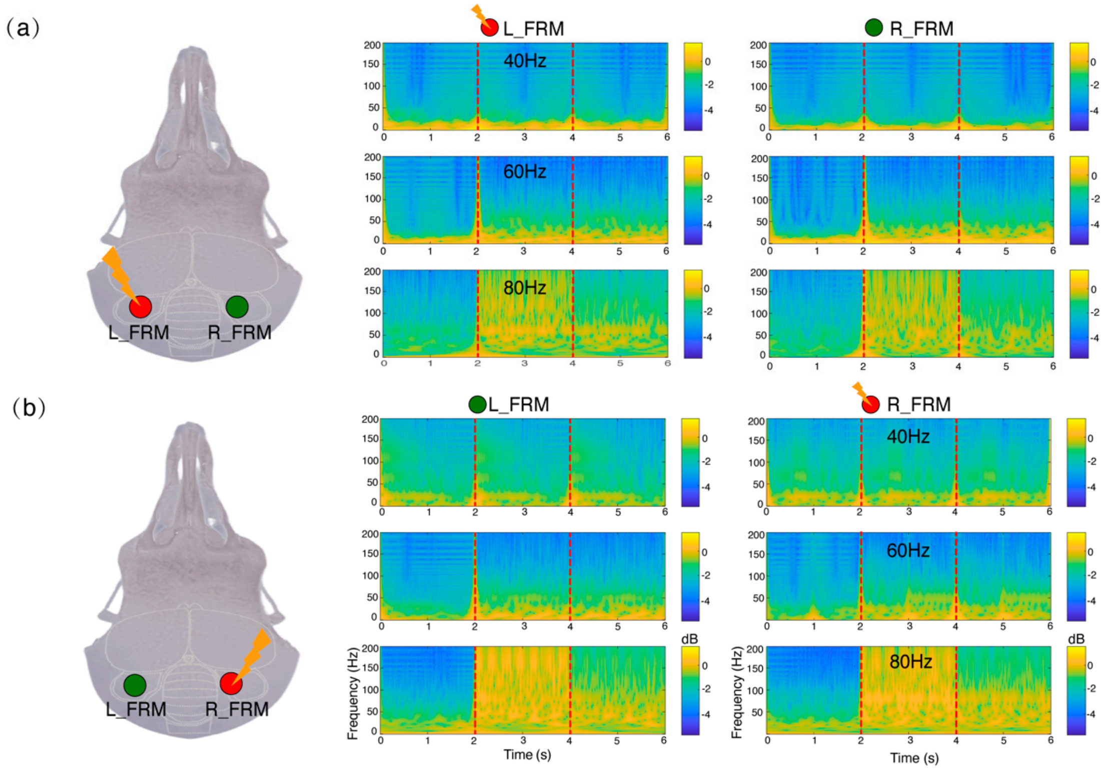

Figure S4. Time-frequency maps of electrically stimulated *formatio reticularis medialis mesencephali* (FRM) nuclei regions and their LFP oscillations on different sides of the pigeon. (a), indicates electrical stimulation of the left FRM nuclei region; (b) electrical stimulation of the right FRM nuclei region.

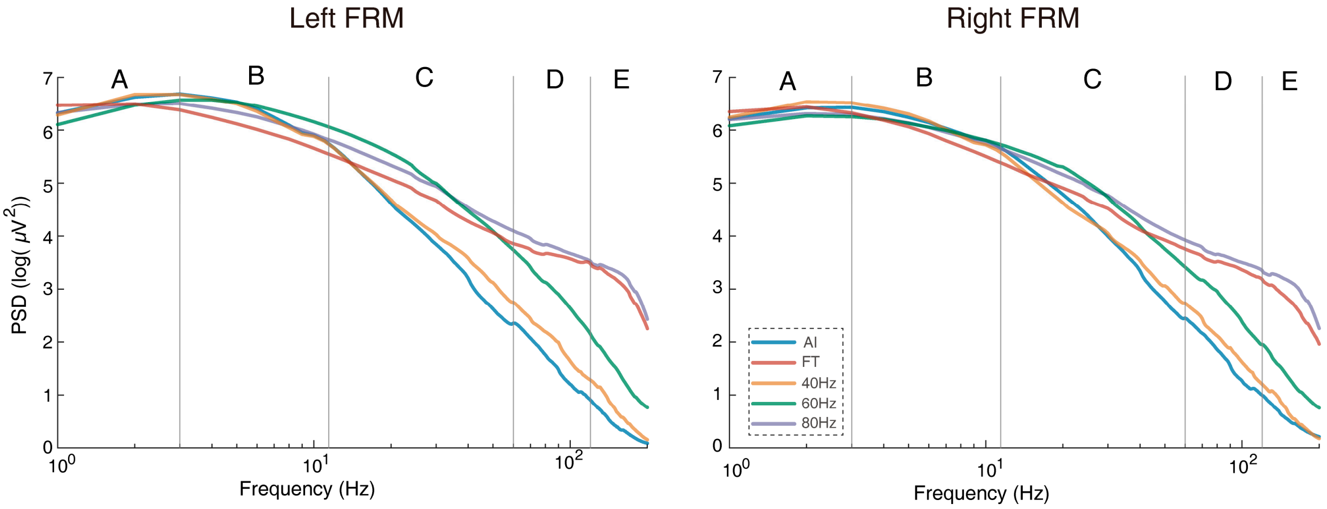

Figure S5. The average waveforms of power spectral density (PSD) for each *formatio reticularis medialis mesencephali* (FRM) nuclei area and each stimulus conditions.

Abbreviation: A, B, C, D, and E represent five different characteristic bands. AI represents pigeons' awake immobility; AT represents pigeons actively turning induced. 40 Hz, 60 Hz, and 80 Hz represent the different stimulus conditions of electrical stimulation.

Table S1. Results of the simple-simple effects analysis between the five different characteristic bands of local field potential (LFP) and the stimulus conditions in *formatio reticularis medialis mesencephali* (FRM) nuclei regions.

Factor	For the Power Spectra $_{(1,11)\ (4,\ 44)\ (4,\ 44)}$ [a]				
	F	ε	p	Partial η^2	LSD
**A**					
Brain area	0.317	1.000	0.584	0.028	N/A
Stimulus	0.095	0.648	0.983	0.009	N/A
2-way interaction	0.137	0.568	0.968	0.012	N/A
**B**					
Brain area	0.399	1.000	0.541	0.035	N/A
Stimulus	20.752	0.738	< 0.001	0.452	AI, 40 Hz > 60 Hz, 80 Hz, AT; 40 Hz > AI
2-way interaction	0.353	0.459	0.841	0.031	N/A
**C**					
Brain area	0.540	1.000	0.478	0.047	N/A
Stimulus	11.229	0.667	< 0.001	0.505	60 Hz, 80 Hz, AT > AI, 40 Hz; 60 Hz > AT, 80 Hz
2-way interaction	0.048	0.438	0.936	0.004	N/A
**D**					
Brain area	0.178	1.000	0.681	0.016	N/A
Stimulus	25.581	0.744	< 0.001	0.699	80 Hz, AT > AI, 40 Hz; 80 Hz > 60 Hz
2-way interaction	0.704	0.409	0.481	0.060	N/A
**E**					
Brain area	0.217	1.000	0.217	0.124	N/A
Stimulus	36.478	0.778	< 0.001	0.752	80 Hz, AT > 60Hz, 40 Hz, AI; 80 Hz > AT; 60Hz > 40 Hz, AI
2-way interaction	0.615	0.233	0.919	0.019	N/A

Note: The superscript symbol "[a]" in the first line of the table denotes the degrees of freedom for the factors "Brain area", "Stimulus" and "Bands", respectively. The numbers in parentheses indicate the values of the degrees of freedom of the factors. F is the F-value from ANOVA, ε the values of epsilon of Greenhouse-Geisser correction, LSD least-significant difference test, A, B, C, D, E represent five different

bands. Abbreviation: AI represents pigeons' awake immobility; AT represents pigeons actively turning induced by food. 40 Hz, 60 Hz, and 80 Hz represent the different frequencies of electrical stimulation. N/A not applicable.
